# Large-Scale Identification of Multiple Classes of Host Defense Peptide-Inducing Compounds for Antimicrobial Therapy

**DOI:** 10.3390/ijms23158400

**Published:** 2022-07-29

**Authors:** Wentao Lyu, Dehui Mi, Paige N. Vinson, Yingping Xiao, Guolong Zhang

**Affiliations:** 1 State Key Laboratory for Managing Biotic and Chemical Threats to the Quality and Safety of Agro-Products, Institute of Agro-Product Safety and Nutrition, Zhejiang Academy of Agricultural Sciences, Hangzhou 310021, China; lvwt@zaas.ac.cn (W.L.); xiaoyp@zaas.ac.cn (Y.X.); 2 Department of Animal and Food Sciences, Oklahoma State University, Stillwater, OK 74078, USA; 3 Vanderbilt High-Throughput Screening Facility, Vanderbilt Institute of Chemical Biology, Nashville, TN 37232, USA; dehui.mi@vanderbilt.edu (D.M.); pvinson@southernresearch.org (P.N.V.)

**Keywords:** host defense peptides, high-throughput screening, host defense peptide inducers, mocetinostat, antimicrobial resistance

## Abstract

The rapid emergence of antibiotic resistance demands new antimicrobial strategies that are less likely to develop resistance. Augmenting the synthesis of endogenous host defense peptides (HDPs) has been proven to be an effective host-directed therapeutic approach. This study aimed to identify small-molecule compounds with a strong ability to induce endogenous HDP synthesis for further development as novel antimicrobial agents. By employing a stable HDP promoter-driven luciferase reporter cell line known as HTC/*AvBD9-luc*, we performed high-throughput screening of 5002 natural and synthetic compounds and identified 110 hits with a minimum Z-score of 2.0. Although they were structurally and functionally diverse, half of these hits were inhibitors of class I histone deacetylases, the phosphoinositide 3-kinase pathway, ion channels, and dopamine and serotonin receptors. Further validations revealed mocetinostat, a benzamide histone deacetylase inhibitor, to be highly potent in enhancing the expression of multiple HDP genes in chicken macrophage cell lines and jejunal explants. Importantly, mocetinostat was more efficient than entinostat and tucidinostat, two structural analogs, in promoting HDP gene expression and the antibacterial activity of chicken macrophages. Taken together, mocetinostat, with its ability to enhance HDP synthesis and the antibacterial activity of host cells, could be potentially developed as a novel antimicrobial for disease control and prevention.

## 1. Introduction

Antimicrobial resistance is a major global healthcare concern [1]. The rapid emergence of antibiotic-resistant pathogens, coupled with a dwindling antibiotic pipeline, demands innovative antimicrobial strategies that are less likely to trigger resistance [1,2]. Host-directed immunotherapies have emerged as promising alternative approaches to disease control and prevention [3,4]. Host defense peptides (HDPs), constitute a large, diverse group of small antimicrobial peptides that act as an important component of innate immunity [5,6]. In vertebrate animals, HDPs are classified into two major families, namely cathelicidins and defensins, that are expressed mainly by phagocytic cells and mucosal epithelial cells [7]. A large array of HDPs are produced in humans and animals to provide the first line of host defense in response to infection and inflammation. For example, humans have one cathelicidin known as LL-37, six α-defensins, and more than 30 β-defensins [7], while four cathelicidins (CATH1-3 and CATHB1) and 14 β-defensins known as AvBD1-14 are produced in chickens [8,9].

HDPs are capable of killing a broad spectrum of pathogens through membrane-lytic mechanisms [7] and, at the same time, exert a profound influence on the regulation of both innate and adaptive immunity by recruiting and promoting the differentiation and activation of different types of immune cell [7,10]. Augmenting the synthesis of endogenous HDPs has become an active host-directed approach to antimicrobial therapy [4,11,12,13]. Besides infectious and inflammatory agents, a number of small-molecule compounds such as butyrate and vitamin D_3_ have been identified as having the ability to induce HDP synthesis in humans and other animals [4,11,12,13]. Some of these HDP-inducing compounds, individually or in combination, show promise in conferring on the hosts an enhanced ability to fight off infections such as shigellosis, tuberculosis, cholera, enteropathogenic *E. coli* diarrhea, necrotic enteritis, and coccidiosis [14,15,16,17,18,19,20,21].

The discovery of HDP-inducing compounds for antimicrobial therapy has become an active area of research. A high-throughput screening (HTS) assay based on a stable *LL-37* promoter-driven luciferase reporter cell line (MN8CampLuc), has been developed and has led to the identification of multiple LL-37 inducers [22,23]. We also established different luciferase reporter cell lines through the stable integration of HDP promoter-driven luciferase reporter genes in macrophages or intestinal epithelial cell lines [24,25], which were subsequently employed to identify a number of HDP-inducing compounds [24,25,26,27]. Here, we report a large-scale screening of 5002 natural and synthetic small-molecule compounds with much greater structural and functional diversities using one such reporter cell line, termed HTC/*AvBD9-luc* [25]. We revealed that histone deacetylase (HDAC) inhibitors are among the most efficacious HDP inducers. We further demonstrated that mocetinostat, being a leading candidate, was highly potent in inducing the expressions of multiple HDP genes and further enhanced the antibacterial activity of host cells. Further characterization of these HDP-inducing compounds may pave the way for their development as novel host-directed immune-boosting antimicrobials.

## 2. Results

### 2.1. HTS for Small-Molecule Compounds That Induce HDP Synthesis

**Figure 1:**
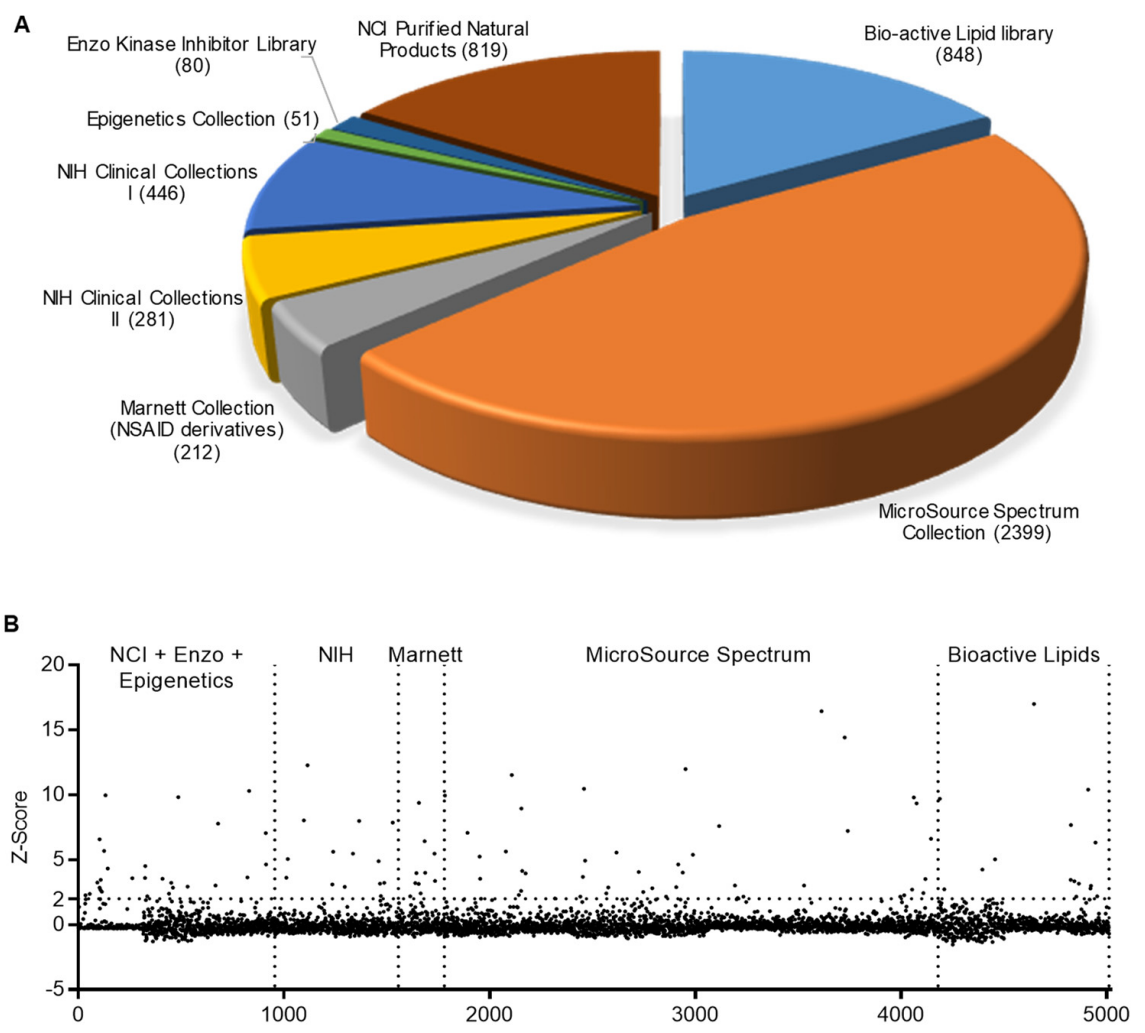
High-throughput screening to identify host defense peptide-inducing compounds. (**A**) Identities of small-molecule compound libraries used in the screening. The number of compounds in each library is shown in the parentheses. (**B**) Z-scores of the 5002 compounds in different libraries. HTC/*AvBD9-luc* luciferase reporter cell line was stimulated in 384-well plates with 20 μM of each compound for 24 h, followed by luciferase and cell viability assays. The Z-scores for each compound were calculated from luciferase activity normalized to cell viability.

To discover additional HDP-inducing compounds, we employed the HTC/*AvBD9-luc* reporter cell line and conducted larger-scale screening of 5002 natural and synthetic small-molecule compounds in several different libraries (Figure 1A). The average Z’ factor [28] across 384-well plates was 0.52 ± 0.03, indicating that the HTS assay was robust and reliable. Based on our preliminary screening and earlier experiences [24,25,26,27], a final concentration of 20 μM was used for each test compound in HTS. Using a Z-score of 2.0 as the threshold [28], we identified 110 compounds, resulting in a hit rate of 2.2% (Figure 1B). These 110 hits were largely scattered across libraries, with a larger percentage being found in the epigenetic compound library (Figure 1B); this is consistent with earlier HTS results, which state that many epigenetic compounds and HDAC inhibitors, in particular, are HDP inducers [24,25,26].

All hits were further compared for their relative *AvDB9*-inducing activity in HTC/*AvBD9-luc* cells at 5, 10, and 20 μM using a luciferase assay. Obviously, most showed increased luciferase activity in at least one concentration used (data not shown). Among the 18 compounds with a minimum 5-fold increase in luciferase activity at 20 μM, each compound dose-dependently induced luciferase activity while causing minimum cytotoxicity, particularly at 5 or 10 μM (Table 1). Approximately half of these hits were known HDAC inhibitors, while three others were involved in the phosphoinositide 3-kinase (PI3K)/protein kinase B (AKT)/mammalian target of rapamycin (mTOR) pathway; this was also observed in an earlier study, as exemplified in wortmannin [25]. The remaining hits included maprotiline hydrochloride, desloratadine, doxorubicin, tetrandrine, quinacrine, and promazine (Table 1), which are norepinephrine reuptake inhibitor, H_1_-antihistamine, topoisomerase II inhibitor, calcium channel blocker, NF-κB inhibitor/p53 activator/histamine N-methyltransferase inhibitor, and D_2_ dopamine receptor inhibitor, respectively [29,30,31,32,33,34,35].

**Figure 2:**
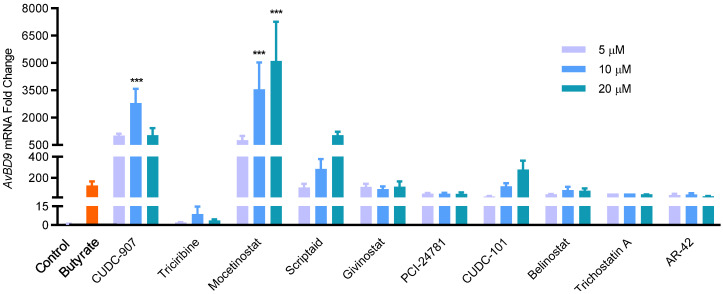
Dose-dependent induction of *AvBD9* mRNA expression in chicken HTC cells in response to ten leading HDP inducers. HTC cells were stimulated in duplicate using different concentrations of each compound for 24 h, followed by RT-qPCR analysis of *AvBD9* mRNA expression. Sodium butyrate (4 mM) was used as a positive control and an equal volume of solvent as a negative control. The results are means ± SEM of three independent experiments. One-way ANOVA was performed, followed by Dunnett’s test. *** *p* < 0.001 (relative to the unstimulated control).

Ten lead compounds with a minimum 20-fold increase in luciferase activity at 20 μM were further selected to compare their relative potency in inducing *AvBD9* mRNA expression in HTC cells using RT-qPCR. As expected, all 10 compounds obviously induced *AvBD9* expression in a dose-dependent manner, and all but triciribine achieved similar efficacy to butyrate (Figure 2). Among them, mocetinostat and CUDC-907 were the most potent, triggering nearly a 1000-fold *AvBD9* induction even at 5 μM, while sodium butyrate produced only a 125-fold increase at the optimal concentration of 4 mM. On the other hand, mocetinostat at 20 μM enhanced *AvBD9* mRNA expression more than 5000-fold (Figure 2). It is worth mentioning that the order of relative potency of individual compounds in the luciferase assay was rather different from the RT-qPCR analysis. For example, although it was among the top compounds in the luciferase reporter assay, givinostat performed much worse than mocetinostat, CUDC-907, and several other compounds in *AvBD9* mRNA induction in HTC cells (Figure 2).

### 2.2. Confirmation of the AvBD9-Inducing Capacity of Mocetinostat in Chicken Macrophages

**Figure 3:**
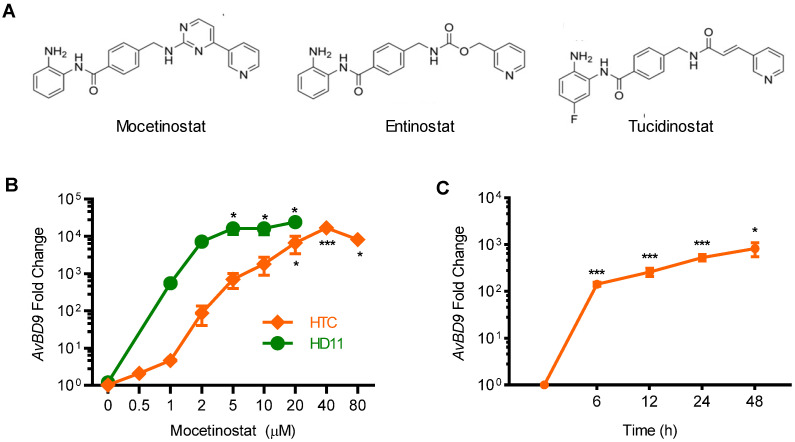
Dose- and time-dependent induction of *AvBD9* mRNA expression by mocetinostat in two different chicken macrophage cell lines. (**A**) Chemical structures of mocetinostat and its two structural analogs, entinostat and tucidinostat. (**B**) Dose-dependent changes in *AvBD9* mRNA expression in chicken HTC and HD11 cell lines in response to different concentrations of mocetinostat for 24 h. (**C**) Time-dependent induction of *AvBD9* mRNA in HTC cells in response to 2 μM mocetinostat for various lengths of time. *AvBD9* mRNA expression levels were evaluated using RT-qPCR. The results are means ± SEM of three independent experiments. One-way ANOVA was performed, followed by Dunnett’s test. * *p* < 0.05, and *** *p* < 0.001 (relative to the unstimulated control).

Mocetinostat, also known as MGCD0103, is a benzamide HDAC inhibitor (Figure 3A) undergoing clinical trials for various forms of cancers [36,37,38,39]. Mocetinostat was the most potent HDP-inducing compound in our assays, and thus, was selected for subsequent characterizations. Mocetinostat was first evaluated for its ability to promote *AvBD9* mRNA expression in a different chicken macrophage cell line, HD11 [40]. As expected, it showed an obvious dose-dependent *AvBD9* induction. In fact, HD11 cells appeared to be more sensitive to mocetinostat than HTC cells (Figure 3B). At each concentration used, a higher magnitude of *AvBD9* gene induction was seen in HD11 cells than in HTC cells. To investigate the kinetics of gene induction, a time-course experiment was conducted in HTC cells in response to 2 μM mocetinostat. An apparent 100-fold increase in *AvBD9* expression was observed as early as 6 h, peaking around 24 h and remaining elevated at 48 h (Figure 3B).

### 2.3. Induction of Multiple HDP and Barrier-Function Genes by Mocetinostat

**Figure 4:**
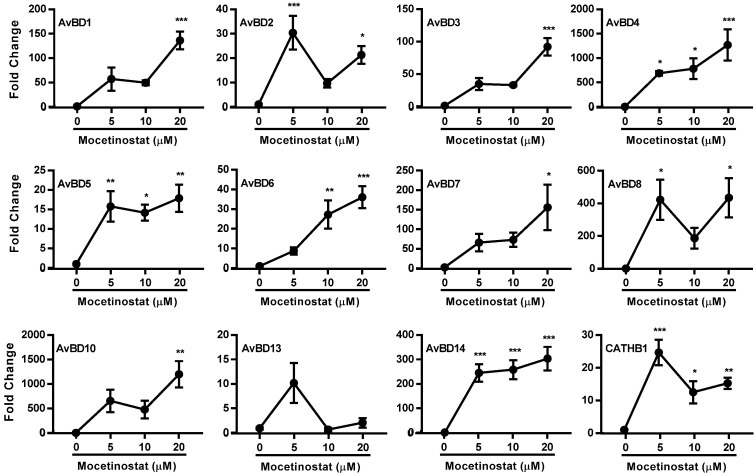
Induction of multiple HDP genes in chicken HTC macrophages in response to mocetinostat. HTC cells were stimulated in duplicate with 5, 10, and 20 μM mocetinostat for 24 h. HDP gene-expression levels were evaluated using RT-qPCR. The results are means ± SEM of three independent experiments. One-way ANOVA was performed, followed by Dunnett’s test. * *p* < 0.05, ** *p* < 0.01, and *** *p* < 0.001 (relative to the unstimulated control).

To verify how other HDPs are regulated by mocetinostat, RT-qPCR was used to analyze the expression levels of multiple chicken HDP mRNAs in HTC cells in response to mocetinostat. It was apparent that mocetinostat enhanced the expression of all HDP genes that are expressed in HTC cells in a dose-dependent manner, although the magnitude of induction varied (Figure 4). For example, like *AvBD9*, *AvBD4* and *AvBD10* were upregulated by 20 μM mocetinostat more than 1000-fold, while *AvBD1, AvBD3*, *AvBD7*, *AvBD8*, and *AvBD14* were increased at least 100-fold. However, the remaining HDP genes showed a peak induction no more than 50-fold in response to mocetinostat (Figure 4).

### 2.4. Comparison of HDP-Inducing Efficacy among Mocetinostat, Entinostat, and Tucidinostat

**Figure 5:**
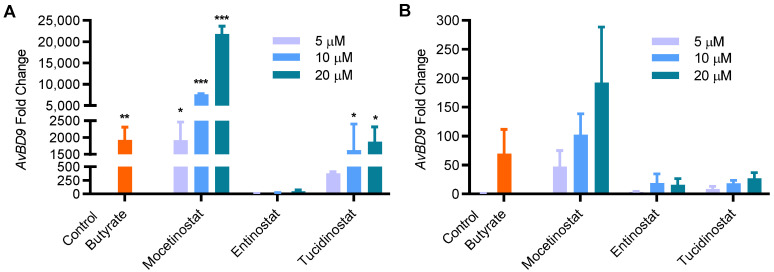
Induction of *AvBD9* mRNA expression by mocetinostat, entinostat, and tucidinostat in HTC cells and chicken jejunal explants. HTC cells (**A**) or chicken jejunal explants (**B**) were exposed to 4 mM butyrate or three different concentrations of mocetinostat, entinostat, and tucidinostat for 24 h, followed by analysis of *AvBD9* gene expression using RT-qPCR. The results are means ± SEM of 2–3 independent experiments. One-way ANOVA was performed, followed by Dunnett’s test. * *p* < 0.05, ** *p* < 0.01, and *** *p* < 0.001 (relative to the unstimulated control).

Entinostat, also known as MS-275 or SNDX-275, was recently identified among the most potent compounds to stimulate human *LL-37* synthesis [23,41]. Tucidinostat, also known as chidamide, was found to have similar efficacy to entinostat in chicken HDP induction in our recent HTS [27]. In fact, both entinostat and tucidinostat are benzamide HDAC inhibitors and structural analogs of mocetinostat (Figure 3A). We sought to directly compare HDP-inducing efficacy among mocetinostat, entinostat, and tucidinostat in chicken HTC cells and jejunal explants. It was apparent that mocetinostat was much more potent in upregulating *AvBD9* mRNA expression in both HTC cells and jejunal explants than entinostat and tucidinostat (Figure 5). For example, 20 μM mocetinostat triggered approximately a 20,000- and 200-fold induction of *AvBD9* mRNA in HTC cells and jejunal explants, respectively, whereas tucidinostat caused approximately an 800-fold *AvBD9* increase in HTC cells and a 25-fold increase in jejunal explants. On the other hand, 20 μM entinostat only produced a less-than-20-fold induction in both cell types (Figure 5).

### 2.5. Mocetinostat Augments the Antibacterial Activity of Chicken Macrophages without Directly Killing Bacteria

**Figure 6:**
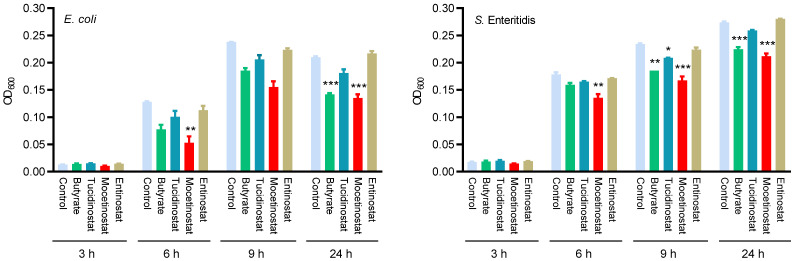
Augmentation of the antibacterial activity of chicken HTC cells by mocetinostat, entinostat, and tucidinostat. Chicken HTCs were stimulated with 10 μM mocetinostat, entinostat, tucidinostat, or 4 mM butyrate for 24 h, followed by cell lysis and incubation of the cell lysate with *Escherichia coli* (ATCC 25,922) or *Salmonella* Enteritidis (ATCC 13,076) for different durations. Bacterial turbidity was measured at OD_600_ as an indication of bacterial growth. The results are means ± SEM of two independent experiments. One-way ANOVA was performed, followed by Dunnett’s test. * *p* < 0.05, ** *p* < 0.01, and *** *p* < 0.001 (relative to the unstimulated control at each time point).

To evaluate whether mocetinostat is capable of boosting the antibacterial activity of host cells through the upregulation of HDP synthesis, HTC cells were treated with mocetinostat, tucidinostat, or entinostat for 24 h, followed by cell lysis and the incubation of cell lysate with *E. coli* (ATCC 26,922) and *Salmonella* Enteritidis (ATCC 13,076) for varying lengths of time using 4 mM butyrate as a positive control. Consistent with our previous studies [25,42], butyrate enhanced the ability of HTC cells to suppress both bacteria. Mocetinostat at 10 μM also significantly augmented bacterial inhibition at 6, 9, and 24 h, while entinostat failed to show significant antibacterial activity at any time point, and tucidinostat showed intermediate activity (Figure 6).

To ensure that augmentation of the antibacterial activity of host cells is not due to the direct antibacterial activity of these compounds, we determined the minimum inhibitory concentrations (MICs) of mocetinostat, entinostat, and tucidinostat using a standard broth microdilution assay [43] using *E. coli* (ATCC 26,922) and *Salmonella* Enteritidis (ATCC 13,076). The MICs of all three compounds were beyond 320 μM, the highest concentration tested, suggesting that they have no obvious antibacterial activity. It is, therefore, likely that HDP inducers such as mocetinostat boost the antibacterial capacity of host cells through the modulation of endogenous HDP synthesis.

## 3. Discussion

A number of small molecules have been found to be capable of inducing HDP synthesis without provoking inflammation [4,11,12,13]. Moreover, HDP inducers are not expected to trigger resistance because they regulate host immunity with no direct interactions with bacteria. Modulating the synthesis of endogenous HDPs is, therefore, being actively explored as an alternative host-directed antimicrobial approach [4,11,12,13]. We have developed a cell-based HTS assay to screen and identify a number of HDP-inducing compounds through several recent small-scale efforts [24,25,26,27]. In this study, we broadened our screening effort to a list of 5002 small-molecule compounds with much greater structural and functional diversity. From primary screening, we identified 110 hits with a minimum Z-score of 2.0. Approximately half of the compounds function as inhibitors of HDACs, the PI3K/AKT/mTOR pathway, dopamine and serotonin receptors, or calcium/sodium channels, with the HDAC and PI3K/AKT/mTOR blockers being among the most efficacious HDP inducers. The other half of the compounds are involved in a variety of other functions.

The top ten hits that produced a minimum 20-fold increase in luciferase activity in HTC/*AvBD9-luc* cells at 20 μM were further confirmed to induce *AvBD9* mRNA expression in a dose-dependent manner in chicken HTC cells, suggesting the validity and effectiveness of our HTS assay. However, we observed a discrepancy among individual compounds in their *AvBD9*-inducing potency between the luciferase and RT-qPCR assays. The reason is likely due to the fact that the luciferase assay is based on the ability of a compound to activate a 2-Kb *AvBD9* gene promoter fragment [25], while RT-qPCR measures the mRNA expression levels of the native *AvBD9* gene. It is possible that some compounds may regulate certain transcription factors that bind beyond the 2-Kb promoter region of the *AvBD9* gene.

Histone acetylation is regulated by the opposing effects of HDACs and histone acetyltransferases (HATs), with the former functioning to remove the acetyl groups from the lysine residues of histones and the latter adding the acetyl group to histones [44]. The balancing act of HDACs and HATs serves to fine-tune chromatin structure, the accessibility of transcriptional factors to their binding sites, and subsequent gene transcription [45]. HDAC inhibitors work by tipping the HDAC/HAT balance, leading to relaxation of the chromatin structure and enhanced gene transcription [46]. Modifying the acetylation status of the promoter has been shown to have a profound impact on the transcription of HDP genes in humans, rats, rabbits, cattle, pigs, and chickens [11]. Therefore, it is, perhaps, not surprising to see that nine out of the 10 most efficacious HDP inducers are HDAC inhibitors, although two (CUDC-907 and CUDC-101) are known to be involved in other functions, as well. These results are in line with our early screening, wherein the most efficient HDP inducers were mostly HDAC inhibitors [24,25,26].

The PI3K/AKT/mTOR pathway is critically involved in cell growth and metabolism [47]. Several inhibitors of this pathway such as wortmannin and CUDC-907 were shown to be potent in HDP induction in this study, which is consistent with our earlier HTS study, in which wortmannin was identified as a top hit [25]. However, several other specific inhibitors to PI3K, AKT, or mTOR were assessed and only had a marginal effect on HDP induction in chicken HTC cells [25]. Additionally, butyrate, a well-known HDAC inhibitor and HDP inducer, was recently shown to enhance mTOR phosphorylation, and knockdown of mTOR significantly reduced butyrate-mediated β-defensin gene expression in mouse intestinal epithelial cells [48]. These lines of apparently conflicting evidence suggest that more studies are needed to implicate the PI3K/AKT/mTOR pathway in HDP synthesis.

Several calcium-channel blockers and serotonin-receptor antagonists were identified as HDP inducers in this study. Consistently, tetrandrine and isotetrandrine, two calcium-channel blockers, and dihydroergocristine mesylate, a serotonin-receptor antagonist, were also identified in our previous screening as top hits [25]. However, the mechanism by which these two classes of compounds induce HDP gene expression is currently unknown, as is the case with the role of dopamine-receptor antagonists in HDP induction.

Although the majority of the hits obtained in the primary screening produced a dose-dependent increase in the follow-up luciferase assay, the luciferase activity of approximately half of the compounds in the secondary screening were not necessarily well-correlated with their Z-score in the primary HTS assay. More than a dozen hits failed to show an obvious increase in luciferase activity in the follow-up experiments. The reason for such a discrepancy is unclear, but could be due to the fact that the compounds used in the secondary screening were obtained from different batches of the libraries or procured from different vendors.

Mocetinostat has been identified as the most potent HDP inducer among all hits identified in this study. It specifically inhibits HDAC1-3 and HDAC11, but with a negligible effect on other HDACs [49]. Mocetinostat causes hyperacetylation of histones and induces apoptosis in cancerous cells; it is currently undergoing clinical trials, either individually or in combination with other epigenetic agents, for several different types of cancer, with varying degrees of success [36,37,38,39]. We have shown that mocetinostat potently induces the expression of multiple HDP genes in different cell types and is more potent than two of its structural and functional analogs, entinostat and tucidinostat, in enhancing HDP synthesis and the antibacterial activity of host cells. Entinostat was identified earlier as a highly potent HDP inducer in humans [23,41], and both entinostat and tucidinostat were recently discovered to be among the most potent epigenetic compounds in HDP induction in chickens [27].

In this study, we revealed that mocetinostat is much more potent than both entinostat and tucidinostat in chickens, which is consistent with their relative efficacy in inhibiting class I HDACs [50]. However, it will be important to evaluate them directly in human and porcine cells to see whether the results in chickens can be extended to other animal species or whether it is only a species-specific observation. It is perhaps the presence of an additional aromatic ring that confers on mocetinostat an improved efficacy in HDP induction. Given its toxicity and limited success in current clinical trials, the application of mocetinostat for disease control and prevention in animals and humans needs to proceed with caution, and further chemical modifications and dosage optimization may be warranted.

It is noted that HDAC inhibitors are potent HDP inducers, but they are also well-known for their anticancer properties [46,51]. Enhanced acetylation of histones, and particularly non-histone proteins, is largely responsible for the antiproliferative/apoptotic effect of HDAC inhibitors. In fact, HDAC inhibition increases the acetylation and activation of multiple proteins involved in cell-cycle arrest and apoptosis such as p53 and Ku70 [46]. However, the mechanism by which HDAC inhibitors induce HDP genes remains largely unknown, although increased histone acetylation has been observed [11]. It is likely that the acetylation of non-histone proteins that are critically involved in HDP gene regulation may play a major role. For example, the activity of several transcription factors that are known to regulate human cathelicidin antimicrobial peptide (*CAMP*) gene expression, including STAT3, HIF-1, and C/EBP, can be directly enhanced by acetylation [52,53,54]. Therefore, it is likely that HDP and antiproliferative/apoptotic genes are regulated independently by different transcription factors that are further subjected to epigenetic modifications, which helps explain the multifaceted function of HDAC inhibitors.

Collectively, we have identified multiple classes of HDP inducers, and further characterization of these compounds has led to the identification of mocetinostat as the most potent. With the potential to enhance HDP synthesis without killing bacteria directly, mocetinostat shows promise for further development as a novel antimicrobial agent for disease control and prevention, with minimal risk of triggering resistance.

## 4. Materials and Methods

### 4.1. Chemicals and Reagents

Cell-culture reagents including RPMI 1640 medium (without phenol red for HTS) and antibiotics (penicillin, streptomycin, puromycin, and gentamicin) were purchased from Lonza (Allendale, NJ, USA), Santa Cruz Biotechnology (Dallas, TX, USA), and Fisher Scientific (Pittsburgh, PA, USA). Fetal bovine serum (FBS) was obtained from Atlanta Biologicals (Flowery Branch, GA, USA). Sodium butyrate was procured from MilliporeSigma (St. Louis, MO, USA), while mocetinostat, entinostat, and tucidinostat were obtained from Cayman Chemical (Ann Arbor, MI, USA). Sodium butyrate was dissolved in RPMI 1640, while all other chemicals were dissolved in dimethyl sulfoxide (DMSO). An equal volume of RPMI 1640 or DMSO was used in all cell-culture experiments as a negative control.

### 4.2. Cell Culture

Chicken HTC [55] and HD11 [40] macrophage cell lines were kind gifts from Dr. Narayan C. Rath and Dr. Hyun S. Lillehoj, respectively, at USDA Agricultural Research Service (ARS). Both cells were cultured in complete RPMI 1640 medium (HyClone, Logan, UT, USA) containing 10% heat-activated fetal bovine serum (Atlanta Biologicals, Flowery Branch, GA, USA), and 100 U/mL penicillin/100 μg/mL streptomycin (Lonza, Walkersville, MD, USA). A stable luciferase reporter cell line, HTC/*AvBD9-luc*, was established and reported earlier through permanent lentiviral integration of the HTC cells with a firefly luciferase gene driven by a 2-Kb promoter of a chicken HDP gene known as avian β-defensin 9 (*AvBD9*) [25]. HTC/*AvBD9-luc* cells were maintained in complete RPMI 1640 supplemented with 0.5 μg/mL puromycin. All cells were incubated at 37 °C and 5% CO_2_ and subcultured every 3–4 days.

### 4.3. HTS for HDP-Inducing Compounds

HTS was conducted in the HTS Facility at the Vanderbilt Institute of Chemical Biology (Nashville, TN, USA). A total of 5002 small-molecule compounds from the MicroSource Spectrum Collection of biologically and structurally diverse compounds (2399), the NIH Clinical Collections I and II of approved and experimental drugs used in human clinical trials (618), the NCI Focused Natural Product Collection (819), the Cayman Bioactive Lipid I Screening Library (823), the Marnett Collection of NSAID derivatives (212), the Enzo Screen-Well^TM^ Kinase Inhibitor Library (80), and the Selleck Chemicals Epigenetics Compound Library (51) were included in the screening (Figure 1A). All compounds were dissolved in DMSO to 10 mM and used in HTS at a final concentration of 20 μM. For primary screening, HTC/*AvBD9-luc* cells were plated at 2 × 10^4^ cells/well in 384-well white tissue-culture plates in 20 μL of complete RPMI 1640 overnight, followed by stimulation with 20 μM of each test compound for 24 h as we previously described [25,26]. Cells that were treated with 4 mM sodium butyrate or left untreated were used as positive and negative controls, respectively. Cell viability was measured using a CellTiter-Blue^®^ Cell Viability Assay kit (Promega, Madison, WI, USA) 4 h before the luciferase assay, which was performed using the Steady-Glo^®^ Luciferase Assay System (Promega, Madison, WI, USA). The relative luciferase activity of each compound was determined after normalization of the cell viability. To evaluate the robustness of the HTS, the Z’-factor [28] was calculated for each plate based on relative luciferase activity of 12 positive and 12 negative controls. For hit selection, the Z-score [28] was calculated for each test compound and a minimum Z-score of 2.0 was considered a hit.

### 4.4. Validation of Hit Compounds

The hits were first confirmed for their relative HDP-inducing potency in HTC/*AvBD9-luc* cells. HTC/*AvBD9-luc* cells were seeded in duplicate in 96-well plates at 4 × 10^4^ cells/well overnight; then, they were treated in duplicate with each hit compound at three different concentrations (5, 10, and 20 μM) for 24 h, followed by cell-viability and luciferase assays. The relative luciferase activity of each compound was determined after normalization of the cell viability. For those with a minimum 20-fold increase in the relative luciferase activity over the unstimulated controls, the compounds were further validated for their ability to induce HDP mRNA expression in parental HTC cells. HTC cells were stimulated in duplicate at 5, 10 and 20 μM in 12-well plates for 24 h; then, they were subjected to RNA isolation and HDP mRNA expression analysis, as described below.

### 4.5. RNA Isolation and RT-qPCR

RNAzol RT (Molecular Research Center, Cincinnati, OH, USA) was used for cell lysis and total RNA isolation. Reverse transcription of total RNA and qPCR were conducted using iSCRIPT RT Supermix (Bio-Rad, Hercules, CA, USA) and iTaq Universal SYBR Green Supermix (Bio-Rad), respectively, as previously described [19,20,21,56]. The mRNA expression levels of different chicken HDP genes were evaluated using gene-specific primers with glyceraldehyde-3-phosphatedehydrogenase (*GAPDH*) as the reference gene, as described previously [19,20,21,56]. The relative fold changes in mRNA gene expression were calculated using the ΔΔCt method [57].

### 4.6. Chicken Intestinal Explant Culture

A 10 cm segment of the jejunum was collected from 1- to 2-week-old broiler chickens, and jejunal explants were prepared immediately after animals were sacrificed, as previously described [25,58]. In brief, after thorough washes of a jejunal segment in cold PBS supplemented with 100 µg/mL of gentamicin, 100 U/mL penicillin, and 100 µg/mL streptomycin, smaller segments (approximately 5 mm × 5 mm) were prepared and placed individually in 12-well plates containing 2 mL RPMI 1640 medium containing 10% FBS, 20 mM HEPES, 100 µg/mL gentamicin, 100 U/mL penicillin, and 100 µg/mL streptomycin; this was followed by the addition of each compound, in triplicate, at different concentrations. The explants were incubated at 37 °C for 24 h in a hypoxia chamber (StemCell Technologies, Vancouver, BC, Canada) flushed with 95% O_2_ and 5% CO_2_. Total RNA isolation and RT-qPCR analysis of chicken HDP gene expression were performed with jejunal explants after stimulation.

### 4.7. Antibacterial Assay of Chicken HTC Macrophages

The influence of HDP-inducing compounds on the antibacterial activity of chicken HTC cells was evaluated as previously described [42,59]. In brief, HTC cells were seeded at 1 × 10^6^ cells/well in 6-well plates in complete RPMI1640 medium. After overnight incubation at 37 °C in 5% CO_2_, cells were stimulated with 4 mM sodium butyrate or 10 μM of the selected compounds for 24 h; this was followed by cell lysis and the incubation of the cell lysate with *Escherichia coli* (ATCC 25922) and *Salmonella enterica* subsp. *enterica* serovar Enteritidis (ATCC 13076), at 2.5 × 10^5^ CFU/mL, in 20% trypticase soy broth containing 1 mM NaH_2_PO_4_ and 25 mM NaHCO_3_ in a 96-well plate at 37 °C. Bacterial growth was monitored at OD_600_ using a SpectraMax M3 (Molecular Devices, Sunnyvale, CA, USA) at 37 °C for 3, 6, 9, and 24 h.

### 4.8. Minimum Inhibitory Concentration (MIC) Assay

A standard broth microdilution assay was used to evaluate the MICs of selected compounds in accordance with the recommendation of the Clinical and Laboratory Standards Institute [43] as previously described [25,60,61,62]. Briefly, *E. coli* (ATCC 25922) and *Salmonella* Enteritidis (ATCC 13076) were streaked onto trypticase soy agar plates. After overnight incubation at 37 °C, 2–3 individual colonies were picked and grown in trypticase soy broth (Thermo Fisher Scientific, Nazareth, PA, USA) at 37 °C for 3 h. The bacteria were diluted in Mueller Hinton Broth (Thermo Fisher Scientific) to 5 × 10^5^ CFU/mL, followed by the addition of 90 μL/well in a 96-well tissue-culture plate. Serially diluted compounds (10 μL) were added to each well in duplicate to final concentrations of 5, 10, 20, 40, 80, 160, and 320 μM. The MIC was defined as the lowest concentration of a compound that produced no visible bacterial growth at 37 °C for 24 h.

### 4.9. Statistical Analysis

The results are expressed as means ± SEM. GraphPad Prism (San Diego, CA, USA) was used to conduct one-way analysis of variance (ANOVA), followed by Dunnett’s test to compare the differences between each treatment and the unstimulated control. Statistical significance was considered as *p* < 0.05.

## Figures and Tables

**Table 1:** Functional properties, Z-scores, fold changes in relative luciferase activity, and cell viability of 18 lead compounds ^1^.

Compound	Major Function	Z-Score	Fold Change			Cell Viability (%)		
			5 μM	10 μM	20 μM	5 μM	10 μM	20 μM
CUDC-907	HDACi/PI3Ki ^2^	9.97	137.04	482.67	264.88	88.1	69.1	51.2
Triciribine	AKTi	3.25	37.36	77.73	156.08	92.8	74.7	79.4
Mocetinostat	HDACi	3.45	21.36	41.72	59.69	96.7	96.0	74.4
Scriptaid	HDACi	4.33	6.17	25.71	50.42	100.3	92.4	80.7
Givinostat	HDACi	5.68	52.38	113.64	46.13	99.1	85.0	92.5
PCI-24781	HDACi	2.85	77.51	82.80	37.43	98.3	87.9	80.9
CUDC-101	HDACi/EGFRi/HER2i	2.54	4.69	12.52	34.17	105.0	66.5	70.3
Belinostat	HDACi	6.58	23.05	41.44	33.96	97.5	98.8	79.7
Trichostatin A	HDACi	17.00	68.08	47.64	27.62	96.1	106.2	68.4
AR-42	HDACi	2.27	107.71	80.82	25.88	103.0	111.2	85.8
Vorinostat	HDACi	3.35	1.59	3.52	14.53	101.3	87.5	74.4
Wortmannin	PI3Ki	2.38	29.61	33.69	12.09	92.4	67.9	58.9
Maprotiline hydrochloride	NRi	5.61	1.53	3.85	7.01	102.3	97.7	64.1
Desloratadine	HH1Ri	3.00	1.73	2.47	6.83	97.6	93.8	76.6
Doxorubicin	TOPIIi	5.64	7.89	101.89	6.56	95.9	71.9	65.8
Tetrandrine	CaChi	2.17	1.22	1.97	6.42	102.3	89.6	69.2
Quinacrine	NF-κBi/p53a/HMTi	3.02	1.78	2.10	6.03	97.7	75.2	60.9
Promazine	D2DRi	2.82	0.64	3.24	5.39	100.4	74.2	58.2

^1^ Z-scores were obtained from the high-throughput screening assay that was conducted once, while the fold changes in the luciferase activity and cell viability were an average of three independent experiments with similar results. ^2^ Abbreviations are listed at the end of the text.

## Data Availability

All data generated during this study are included in this published article.

## Abbreviation

AKTi	protein kinase B inhibitor
AvBD9	avian β-defensin 9
CaChi	calcium-channel inhibitor
CLDN1	claudin 1
D2DRi	dopamine receptor D_2_ inhibitor
EGFRi	epidermal growth factor receptor antagonist
GAPDH	glyceraldehyde-3-phosphate dehydrogenase
HDAC	histone deacetylase
HDACi	HDAC inhibitor
HDPs	host defense peptides
HER2	human epidermal growth factor receptor 2 antagonist
HH1Ri	histamine H1 receptor inhibitor
HMT	histone methyltransferase inhibitor
HMT	HMT inhibitor
HTS	high-throughput screening
MIC	minimum inhibitory concentration
mTOR	mammalian target of rapamycin
MUC2	mucin 2
NF-κBi	inhibitor to nuclear factor kappa-light-chain-enhancer of activated B cells
NRi	noradrenaline reuptake inhibitor
p53a	p53 activator
PBMCs	peripheral blood mononuclear cells
PI3Ki	phosphatidylinositide 3-kinase inhibitor
RT-qPCR	reverse transcriptase-quantitative PCR
SEM	standard error of the mean
TJP1	tight junction protein 1
TOPIIi	topoisomerase II inhibitor

## References

[1] Schrader S.M., Vaubourgeix J., Nathan C. (2020). Biology of antimicrobial resistance and approaches to combat it. Sci. Transl. Med..

[2] Ghosh C., Sarkar P., Issa R., Haldar J. (2019). Alternatives to Conventional Antibiotics in the Era of Antimicrobial Resistance. Trends Microbiol..

[3] Zumla A., Rao M., Wallis R.S., Kaufmann S.H., Rustomjee R., Mwaba P., Vilaplana C., Yeboah-Manu D., Chakaya J., Ippolito G. (2016). Host-directed therapies for infectious diseases: Current status, recent progress, and future prospects. Lancet Infect. Dis..

[4] Bergman P., Raqib R., Rekha R.S., Agerberth B., Gudmundsson G.H. (2020). Host Directed Therapy against Infection by Boosting Innate Immunity. Front. Immunol..

[5] Ting D.S.J., Beuerman R.W., Dua H.S., Lakshminarayanan R., Mohammed I. (2020). Strategies in Translating the Therapeutic Potentials of Host Defense Peptides. Front. Immunol..

[6] Magana M., Pushpanathan M., Santos A.L., Leanse L., Fernandez M., Ioannidis A., Giulianotti M.A., Apidianakis Y., Bradfute S., Ferguson A.L. (2020). The value of antimicrobial peptides in the age of resistance. Lancet Infect. Dis..

[7] Mookherjee N., Anderson M.A., Haagsman H.P., Davidson D.J. (2020). Antimicrobial host defence peptides: Functions and clinical potential. Nat. Rev. Drug Discov..

[8] Zhang G., Sunkara L.T. (2014). Avian antimicrobial host defense peptides: From biology to therapeutic applications. Pharmaceuticals.

[9] Cuperus T., Coorens M., van Dijk A., Haagsman H.P. (2013). Avian host defense peptides. Dev. Comp. Immunol..

[10] Hancock R.E., Haney E.F., Gill E.E. (2016). The immunology of host defence peptides: Beyond antimicrobial activity. Nat. Rev. Immunol..

[11] Rodriguez-Carlos A., Jacobo-Delgado Y.M., Santos-Mena A.O., Rivas-Santiago B. (2021). Modulation of cathelicidin and defensins by histone deacetylase inhibitors: A potential treatment for multi-drug resistant infectious diseases. Peptides.

[12] Wu J., Ma N., Johnston L.J., Ma X. (2020). Dietary Nutrients Mediate Intestinal Host Defense Peptide Expression. Adv. Nutr..

[13] Robinson K., Ma X., Liu Y., Qiao S., Hou Y., Zhang G. (2018). Dietary modulation of endogenous host defense peptide synthesis as an alternative approach to in-feed antibiotics. Anim. Nutr..

[14] Raqib R., Sarker P., Bergman P., Ara G., Lindh M., Sack D.A., Nasirul Islam K.M., Gudmundsson G.H., Andersson J., Agerberth B. (2006). Improved outcome in shigellosis associated with butyrate induction of an endogenous peptide antibiotic. Proc. Natl. Acad. Sci. USA.

[15] Al-Mamun A., Mily A., Sarker P., Tiash S., Navarro A., Akter M., Talukder K.A., Islam M.F., Agerberth B., Gudmundsson G.H. (2013). Treatment with phenylbutyrate in a pre-clinical trial reduces diarrhea due to enteropathogenic Escherichia coli: Link to cathelicidin induction. Microbes Infect..

[16] Mily A., Rekha R.S., Kamal S.M., Arifuzzaman A.S., Rahim Z., Khan L., Haq M.A., Zaman K., Bergman P., Brighenti S. (2015). Significant Effects of Oral Phenylbutyrate and Vitamin D3 Adjunctive Therapy in Pulmonary Tuberculosis: A Randomized Controlled Trial. PLoS ONE.

[17] Sarker P., Ahmed S., Tiash S., Rekha R.S., Stromberg R., Andersson J., Bergman P., Gudmundsson G.H., Agerberth B., Raqib R. (2011). Phenylbutyrate counteracts Shigella mediated downregulation of cathelicidin in rabbit lung and intestinal epithelia: A potential therapeutic strategy. PLoS ONE.

[18] Sarker P., Banik A., Stromberg R., Gudmundsson G.H., Raqib R., Agerberth B. (2017). Treatment with Entinostat Heals Experimental Cholera by Affecting Physical and Chemical Barrier Functions of Intestinal Epithelia. Antimicrob. Agents Chemother..

[19] Yang Q., Whitmore M.A., Robinson K., Lyu W., Zhang G. (2021). Butyrate, Forskolin, and Lactose Synergistically Enhance Disease Resistance by Inducing the Expression of the Genes Involved in Innate Host Defense and Barrier Function. Antibiotics.

[20] Robinson K., Yang Q., Li H., Zhang L., Aylward B., Arsenault R.J., Zhang G. (2021). Butyrate and Forskolin Augment Host Defense, Barrier Function, and Disease Resistance without Eliciting Inflammation. Front. Nutr..

[21] Yang Q., Chen B., Robinson K., Belem T., Lyu W., Deng Z., Ramanathan R., Zhang G. (2022). Butyrate in combination with forskolin alleviates necrotic enteritis, increases feed efficiency, and improves carcass composition of broilers. J. Anim. Sci. Biotechnol..

[22] Nylen F., Miraglia E., Cederlund A., Ottosson H., Stromberg R., Gudmundsson G.H., Agerberth B. (2014). Boosting innate immunity: Development and validation of a cell-based screening assay to identify LL-37 inducers. Innate Immun..

[23] Ottosson H., Nylen F., Sarker P., Miraglia E., Bergman P., Gudmundsson G.H., Raqib R., Agerberth B., Stromberg R. (2016). Potent Inducers of Endogenous Antimicrobial Peptides for Host Directed Therapy of Infections. Sci. Rep..

[24] Deng Z., Wang J., Lyu W., Wieneke X., Matts R., Ma X., Zhang G. (2018). Development of a Cell-Based High-Throughput Screening Assay to Identify Porcine Host Defense Peptide-Inducing Compounds. J. Immunol. Res..

[25] Lyu W., Deng Z., Sunkara L.T., Becker S., Robinson K., Matts R., Zhang G. (2018). High Throughput Screening for Natural Host Defense Peptide-Inducing Compounds as Novel Alternatives to Antibiotics. Front. Cell. Infect. Microbiol..

[26] Wang J., Lyu W., Zhang W., Chen Y., Luo F., Wang Y., Ji H., Zhang G. (2021). Discovery of natural products capable of inducing porcine host defense peptide gene expression using cell-based high throughput screening. J. Anim. Sci. Biotechnol..

[27] Deng Z., Lyu W., Zhang G. (2022). High-Throughput Identification of Epigenetic Compounds to Enhance Chicken Host Defense Peptide Gene Expression. Antibiotics.

[28] Zhang J.H., Chung T.D., Oldenburg K.R. (1999). A Simple Statistical Parameter for Use in Evaluation and Validation of High Throughput Screening Assays. J. Biomol. Screen..

[29] Harper B., Hughes I.E. (1977). A comparison in rabbit isolated hearts of the dysrhythmogenic potential of amitriptyline, maprotiline and mianserin in relation to their ability to block noradrenaline uptake. Br. J. Pharmacol..

[30] Canonica G.W., Blaiss M. (2011). Antihistaminic, anti-inflammatory, and antiallergic properties of the nonsedating second-generation antihistamine desloratadine: A review of the evidence. World Allergy Organ. J..

[31] Pommier Y., Leo E., Zhang H., Marchand C. (2010). DNA topoisomerases and their poisoning by anticancer and antibacterial drugs. Chem. Biol..

[32] King V.F., Garcia M.L., Himmel D., Reuben J.P., Lam Y.K., Pan J.X., Han G.Q., Kaczorowski G.J. (1988). Interaction of tetrandrine with slowly inactivating calcium channels. Characterization of calcium channel modulation by an alkaloid of Chinese medicinal herb origin. J. Biol. Chem..

[33] Gurova K.V., Hill J.E., Guo C., Prokvolit A., Burdelya L.G., Samoylova E., Khodyakova A.V., Ganapathi R., Ganapathi M., Tararova N.D. (2005). Small molecules that reactivate p53 in renal cell carcinoma reveal a NF-kappaB-dependent mechanism of p53 suppression in tumors. Proc. Natl. Acad. Sci. USA.

[34] Horton J.R., Sawada K., Nishibori M., Zhang X., Cheng X. (2001). Two polymorphic forms of human histamine methyltransferase: Structural, thermal, and kinetic comparisons. Structure.

[35] Creese I., Burt D.R., Snyder S.H. (1976). Dopamine receptor binding predicts clinical and pharmacological potencies of antischizophrenic drugs. Science.

[36] Blum K.A., Advani A., Fernandez L., Van Der Jagt R., Brandwein J., Kambhampati S., Kassis J., Davis M., Bonfils C., Dubay M. (2009). Phase II study of the histone deacetylase inhibitor MGCD0103 in patients with previously treated chronic lymphocytic leukaemia. Br. J. Haematol..

[37] Batlevi C.L., Crump M., Andreadis C., Rizzieri D., Assouline S.E., Fox S., van der Jagt R.H.C., Copeland A., Potvin D., Chao R. (2017). A phase 2 study of mocetinostat, a histone deacetylase inhibitor, in relapsed or refractory lymphoma. Br. J. Haematol..

[38] Grivas P., Mortazavi A., Picus J., Hahn N.M., Milowsky M.I., Hart L.L., Alva A., Bellmunt J., Pal S.K., Bambury R.M. (2019). Mocetinostat for patients with previously treated, locally advanced/metastatic urothelial carcinoma and inactivating alterations of acetyltransferase genes. Cancer.

[39] Choy E., Ballman K., Chen J., Dickson M.A., Chugh R., George S., Okuno S., Pollock R., Patel R.M., Hoering A. (2018). SARC018_SPORE02: Phase II Study of Mocetinostat Administered with Gemcitabine for Patients with Metastatic Leiomyosarcoma with Progression or Relapse following Prior Treatment with Gemcitabine-Containing Therapy. Sarcoma.

[40] Beug H., von Kirchbach A., Doderlein G., Conscience J.F., Graf T. (1979). Chicken hematopoietic cells transformed by seven strains of defective avian leukemia viruses display three distinct phenotypes of differentiation. Cell.

[41] Miraglia E., Nylen F., Johansson K., Arner E., Cebula M., Farmand S., Ottosson H., Stromberg R., Gudmundsson G.H., Agerberth B. (2016). Entinostat up-regulates the CAMP gene encoding LL-37 via activation of STAT3 and HIF-1alpha transcription factors. Sci. Rep..

[42] Sunkara L.T., Achanta M., Schreiber N.B., Bommineni Y.R., Dai G., Jiang W., Lamont S., Lillehoj H.S., Beker A., Teeter R.G. (2011). Butyrate enhances disease resistance of chickens by inducing antimicrobial host defense peptide gene expression. PLoS ONE.

[43] National Committee for Clinical Laboratory Standards (2003). Methods for Dilution Antimicrobial Susceptibility Tests for Bacteria that Grow Aerobically.

[44] Shvedunova M., Akhtar A. (2022). Modulation of cellular processes by histone and non-histone protein acetylation. Nat. Rev. Mol. Cell. Biol..

[45] Barnes C.E., English D.M., Cowley S.M. (2019). Acetylation & Co: An expanding repertoire of histone acylations regulates chromatin and transcription. Essays Biochem..

[46] Eckschlager T., Plch J., Stiborova M., Hrabeta J. (2017). Histone Deacetylase Inhibitors as Anticancer Drugs. Int. J. Mol. Sci..

[47] Zughaibi T.A., Suhail M., Tarique M., Tabrez S. (2021). Targeting PI3K/Akt/mTOR Pathway by Different Flavonoids: A Cancer Chemopreventive Approach. Int. J. Mol. Sci..

[48] Zhao Y., Chen F., Wu W., Sun M., Bilotta A.J., Yao S., Xiao Y., Huang X., Eaves-Pyles T.D., Golovko G. (2018). GPR43 mediates microbiota metabolite SCFA regulation of antimicrobial peptide expression in intestinal epithelial cells via activation of mTOR and STAT3. Mucosal. Immunol..

[49] Zhou N., Moradei O., Raeppel S., Leit S., Frechette S., Gaudette F., Paquin I., Bernstein N., Bouchain G., Vaisburg A. (2008). Discovery of N-(2-aminophenyl)-4-[(4-pyridin-3-ylpyrimidin-2-ylamino)methyl]benzamide (MGCD0103), an orally active histone deacetylase inhibitor. J. Med. Chem..

[50] Khan N., Jeffers M., Kumar S., Hackett C., Boldog F., Khramtsov N., Qian X., Mills E., Berghs S.C., Carey N. (2008). Determination of the class and isoform selectivity of small-molecule histone deacetylase inhibitors. Biochem. J..

[51] Ho T.C.S., Chan A.H.Y., Ganesan A. (2020). Thirty Years of HDAC Inhibitors: 2020 Insight and Hindsight. J. Med. Chem..

[52] Lee J.L., Wang M.J., Chen J.Y. (2009). Acetylation and activation of STAT3 mediated by nuclear translocation of CD44. J. Cell Biol..

[53] Albanese A., Daly L.A., Mennerich D., Kietzmann T., See V. (2020). The Role of Hypoxia-Inducible Factor Post-Translational Modifications in Regulating Its Localisation, Stability, and Activity. Int. J. Mol. Sci..

[54] Cesena T.I., Cardinaux J.R., Kwok R., Schwartz J. (2007). CCAAT/enhancer-binding protein (C/EBP) beta is acetylated at multiple lysines: Acetylation of C/EBPbeta at lysine 39 modulates its ability to activate transcription. J. Biol. Chem..

[55] Rath N.C., Parcells M.S., Xie H., Santin E. (2003). Characterization of a spontaneously transformed chicken mononuclear cell line. Vet. Immunol. Immunopathol..

[56] Yang Q., Fong L.A., Lyu W., Sunkara L.T., Xiao K., Zhang G. (2021). Synergistic Induction of Chicken Antimicrobial Host Defense Peptide Gene Expression by Butyrate and Sugars. Front. Microbiol..

[57] Schmittgen T.D., Livak K.J. (2008). Analyzing real-time PCR data by the comparative C(T) method. Nat. Protoc..

[58] Sunkara L.T., Zeng X., Curtis A.R., Zhang G. (2014). Cyclic AMP synergizes with butyrate in promoting beta-defensin 9 expression in chickens. Mol. Immunol..

[59] Schauber J., Dorschner R.A., Yamasaki K., Brouha B., Gallo R.L. (2006). Control of the innate epithelial antimicrobial response is cell-type specific and dependent on relevant microenvironmental stimuli. Immunology.

[60] Xiao Y., Dai H., Bommineni Y.R., Soulages J.L., Gong Y.X., Prakash O., Zhang G. (2006). Structure-activity relationships of fowlicidin-1, a cathelicidin antimicrobial peptide in chicken. FEBS J..

[61] Xiao Y., Herrera A.I., Bommineni Y.R., Soulages J.L., Prakash O., Zhang G. (2009). The central kink region of fowlicidin-2, an alpha-helical host defense peptide, is critically involved in bacterial killing and endotoxin neutralization. J. Innate Immun..

[62] Bommineni Y.R., Dai H., Gong Y.X., Soulages J.L., Fernando S.C., Desilva U., Prakash O., Zhang G. (2007). Fowlicidin-3 is an alpha-helical cationic host defense peptide with potent antibacterial and lipopolysaccharide-neutralizing activities. FEBS J..

